# P-1559. Investigating the Effect of HLA-DRB1 Variants on T cell Response as a Contributing Risk Factor for Staphylococcus aureus Bacteremia

**DOI:** 10.1093/ofid/ofaf695.1739

**Published:** 2026-01-11

**Authors:** Priscilla La, Jackson L Kair, Yazhong Tao, Ashley Drabik, Timothy Davenport, Daniel Dorfsman, William K Scott, Derek M Dykxhoorn, Felicia Ruffin, Vance G Fowler, Annette M Jackson

**Affiliations:** Duke University, Durham, North Carolina; University of Copenhagen, Fairfax, Virginia; Duke University, Durham, North Carolina; Duke University, Durham, North Carolina; Duke University, Durham, North Carolina; University of Miami Hussman Institute for Human Genetics, Miami, Florida; University of Miami, Miami, Florida; University of Miami, Miami, Florida; Duke University Medical Center, Durham, NC; Duke University Medical Center, Durham, NC; Duke University, Durham, North Carolina

## Abstract

**Background:**

The impact of host genetic variability on *Staphylococcus aureus* bacteremia (SAB) risk is unknown. In genome-wide association studies, we identified specific HLA-class II variants associated with higher risk (HLA-DRB1*04:01 [OR = 1.121 (0.952, 1.321)] & 03:01 [OR = 1.103 (0.960, 1.269)] or lower risk (HLA-DRB1*07:01) of SAB. The aim of this study is to determine how HLA-DRB1 variants, which differ in *S. aureus* peptide presentation, may also differ in their ability to activate T cells and therefore protective immunity.

**Figure:**
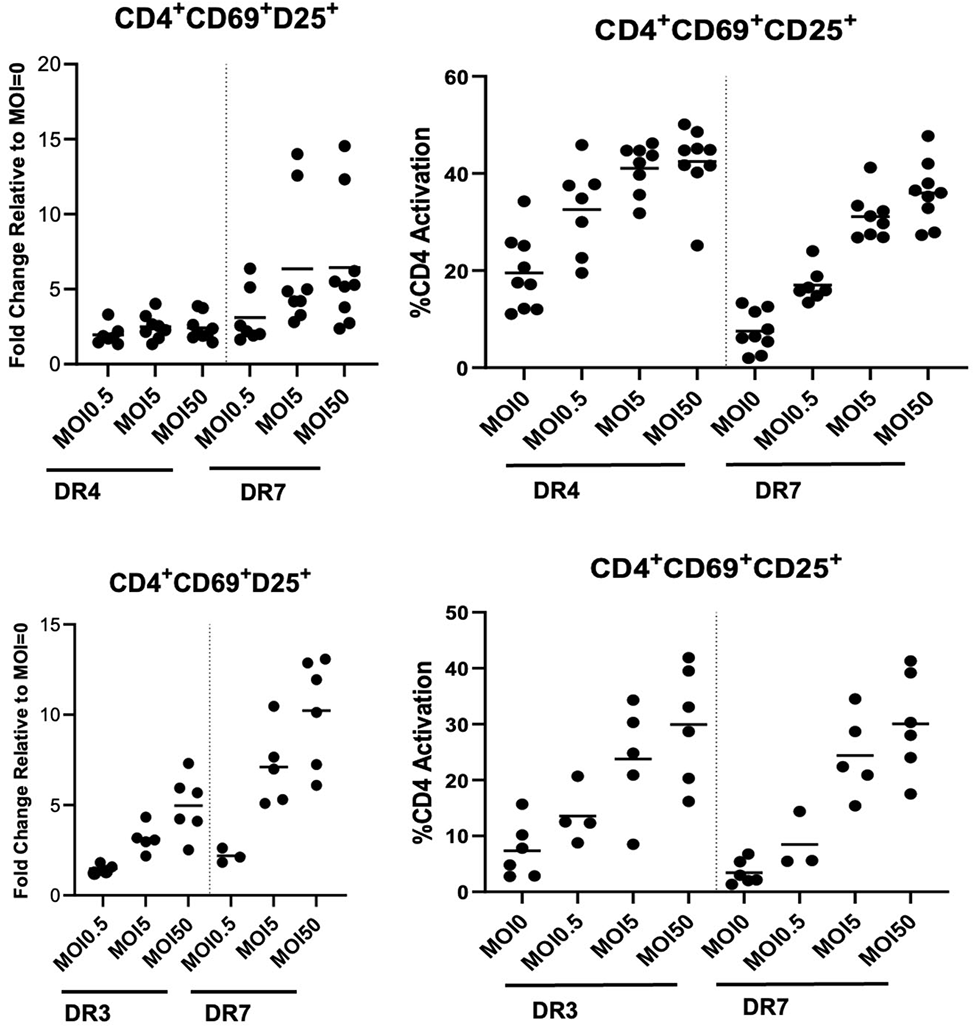

**Fig. 2:**
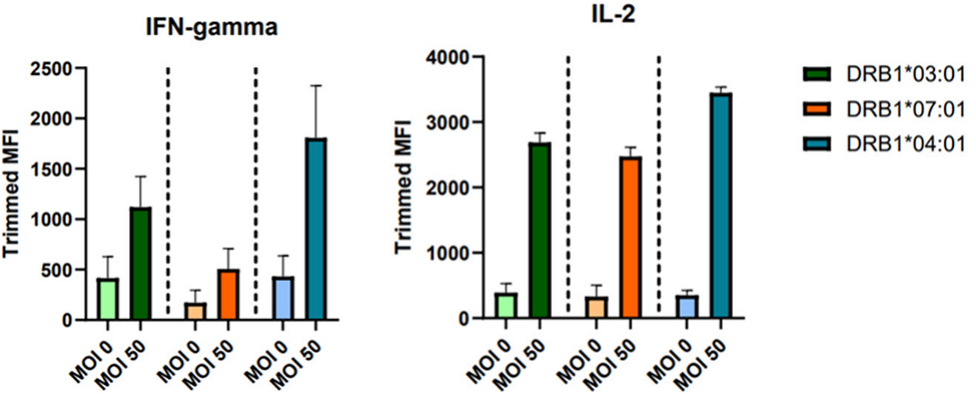
Cytokine production by MOI and HLA-DRB1 variants

**Methods:**

To measure differential CD4+ T cell responses elicited by HLA-class II variation, CD4 T cells isolated from healthy donor HLA-DRB1 heterozygous peripheral blood mononuclear cells were split and separately co-cultured for 72 hrs with homozygous HLA-DRB1 matched B- lymphoblastoid cell lines that were unchallenged or pulsed overnight with dead *S. aureus* (USA 300) across a range of concentrations (multiplicity of infection [MOI = 0.5, 5, 50]). CD4 T cell activation (CD69+ CD25+) was assessed by flow cytometry (BD Fortessa & FlowJo) using monoclonal antibodies specific for CD4 PerCP-Cy5.5, CD69-PE, and CD25-APC and expressed as mean and standard deviation (Prism). Culture supernatants were collected for cytokine quantification (ProcartaPlex) using the Luminex 200 analyzer (xPONENT). Comparisons between MOI and HLA-DRB1 allele were determined with a two-way ANOVA (Prism).

**Results:**

We observed variable T cell responses to *S. aureus* across different HLA-DRB1 alleles. HLA-DRB1*07:01 elicited a greater fold change in CD4+ T cell activation (MOI 0.5= 2.84 ±1.5; MOI 5= 6.65 ±3.6; MOI 50= 7.96 ±4.1) compared to HLA-DRB1*04:01 (MOI 0.5= 1.48 ±0.27; MOI 5= 3.15 ±0.77; MOI 50= 4.96 ±1.7) or 03:01 (MOI 0.5 =1.95 ±0.66; MOI 5= 2.56 ±0.81; MOI 50= 2.42 ±0.86) across *S. aureus* concentrations. Variable CD4 T cell cytokine response to recognition of *S*. *aureus* peptide was also observed across HLA-DRB1*04:01, 03:01, and 07:01 variants. Cytokines associated with Th1 response and T cell proliferation are shown, but these markers did not reach statistical significance.

**Conclusion:**

We provide *in vitro* evidence to suggest that HLA-DRB1 variation in *S. aureus* peptide presentation impacts CD4+ T cell activation and may explain the association between certain HLA-DRB1 haplotypes and SAB.

**Disclosures:**

Vance G. Fowler, MD, MHS, Affinergy, Janssen, Contrafect: Advisor/Consultant|AstraZeneca; EDE; Basilea: Grant/Research Support|Debiopharm, GSK; Affinium, Basilea,: Advisor/Consultant|Destiny, Amphliphi, Armata, Akagera: Advisor/Consultant|Merck; Contrafect; Karius; Janssen: Grant/Research Support|UpToDate: Royalties|Valanbio: Stock options

